# CADASIL: case report

**DOI:** 10.1590/S1980-57642012DN06030013

**Published:** 2012

**Authors:** Julio Cesar Vasconcelos da Silva, Emerson L. Gasparetto, Eliasz Engelhardt

**Affiliations:** 1 Neuropsicólogo, Mestre em Clínica Médica/Neurologia-UFRJ, Aluno de Doutorado-CDA/IPUB, Universidade Federal do Rio de Janeiro, Rio de Janeiro RJ, Brazil; 2 Professor Adjunto, Departamento de Radiologia, Faculdade de Medicina, Universidade Federal do Rio de Janeiro, Rio de Janeiro RJ, Brazil; 3 Setor de Neurologia Cognitiva e do Comportamento-INDC-CDA/IPUB, Universidade Federal do Rio de Janeiro, Rio de Janeiro RJ, Brazil

**Keywords:** CADASIL, Notch3, cognition, neuropsychology

## Abstract

Cerebral Autosomal Dominant Arteriopathy with Subcortical Infarcts and Leukoencephalopathy (CADASIL) is a hereditary cerebral arteriopathy caused by mutations in the Notch-3 gene. The diagnosis is reached by skin biopsy revealing presence of granular osmiophílic material (GOM), and/or by genetic testing for Notch-3. We report a case of a 52-year-old man with recurrent transient ischemic attacks (TIA), migraine, in addition to progressive sensory, motor and cognitive impairment. He was submitted to a neuropsychological assessment with the CERAD (Consortium to Establish a Registry for Alzheimer's Disease) battery along with other tests, as well as neuroimaging and genetic analysis for Notch-3, confirming the diagnosis. Executive function, memory, language and important apraxic changes were found. Imaging studies suggested greater involvement in the frontal lobes and deep areas of the brain.

## Abstract (pt)

*Cerebral Autosomal Dominant Arteriopathy with Subcortical Infarcts and Leukoencephalopathy* (CADASIL) é uma arteriopatia cerebral hereditária causada por mutações no gene Notch-3. O diagnóstico é feito através de biópsia da pele onde se verifica presença de material osmiofílico granular (GOM) e/ou por teste genético para Notch-3. é relatado o caso de um homem de 52 anos, com ataques isquêmicos transitórios (AIT) recorrentes, enxaqueca e comprometimento sensitivo e motor, e cognitivo progressivo. Foi submetido à avaliação neuropsicológica através da bateria CERAD (Consortium to Establish a Registry for Alzheimer's Disease) e outros testes, exames de neuroimagem e análise genética para Notch-3, que confirmou o diagnóstico. Foram encontradas alterações em função executiva, memória, linguagem e importante comprometimento apráxico. Exames de imagem sugerem um maior envolvimento dos lobos frontais e áreas profundas do cérebro.

## INTRODUCTION

Cerebral Autosomal Dominant Arteriopathy with Subcortical Infarcts and Leukoencephalopathy (CADASIL) is a hereditary early-onset vascular disease causing recurrent ischemic subcortical infarcts, generally accompanied by migraine, cognitive impairment, psychiatric symptoms and progressively severe neurologic deficits.^1,2^

Several methods for diagnosing CADASIL have been proposed. The first Magnetic Resonance Imaging (MRI) characteristics of CADASIL were described in 1991.^3,4^ Generally, they reveal areas of T1 hypointensity and hyperintensity on T2 and FLAIR (Fluid Attenuation Inversion Recovery) images in subcortical white matter, initially affecting temporal lobes and external capsules and spreading to other regions, as well as the presence of lacunar infarcts.^5,6^ Practically all patients manifest the condition before the age of 60 years, while changes on MRI have been detected in individuals younger than 35 years.^4^ In addition, the presence of Granular Osmiophilic Material (GOM) in capillary blood vessels of the skin and muscle on biopsy and genetic studies (Notch 3 analysis) play a key diagnostic role. Biopsy exams have high specificity (up to 100%) yet low sensitivity (less than 50%). Notch 3 testing has been proposed as the primary diagnostic approach, allowing the detection of 90% of affected individuals.^3^

## CASE REPORT

A 52-year-old man, right-handed, with ten years of schooling and positive family history for CADASIL, was attended at our service in 2008. He is both hypertensive and diabetic. The patient presented with a blood pressure (BP) of 200 × 120 mm/Hg and glycemia of 800 mg/d at the first stroke episode. Currently, he is in use of the medications Co-Renitec and Amaryl D 4 mg, controlling both BP and glycemia at normal levels.

The presence of these risk factors makes this case of special interest, showing the importance diagnostic confirmation by genotyping, with regard to differential diagnosis.

The disease initially manifested with transitory ischemic attacks (TIA) followed by sensitivity symptoms (paresthesia) and motor signs (faciobrachiocrural hemiparesis) to the left side. The patient reported episodes of migraine preceded by visual aura. Clinical evolution was rapid and progressive with the emergence of cognitive impairment and worsening motor picture.

Neuropsychological assessment was carried out by applying the Consortium to Establish a Registry for Alzheimer's Disease (CERAD)^8^ battery, which includes the Mini-Mental State Examination (MMSE) validated for use in Brazil^9^ and complementary tests focusing on executive function - the Trail-Making Tests (A and B) (TMT-A and TMT-B),^10^ the Clock Completion Test (CCT) (maximum errors: 7, normal: 0 to 3 and abnormal: 4 to 7),^11^ the Complex Figure Copying Test,^12^ Gesture Imitation^12^ and the version of the Clinical Dementia Rating (CDR)^13^ scale validated for use in Brazil.^14^ No formal test was applied to assess functional independence. However, an interview focusing on occupational aspects and activities of daily living (ADL), including the conducting and handling of personal finances, was conducted with the patient reporting no significant functional problems, a finding corroborated by at least one family member.

The results of the neuropsychological assessment showed changes, as shown in Table 1.

**Table 1:** Results of CERAD neuropsychological assessment and supplementary tests.

Tests	Scores	ND (m±sd)^9^
MMSE	24	27.8±2.2
Verbal Fluency (animals)	9	15.6±3.9
Boston Naming	9	13.1±1.7
Words list - register/learning	12	18.0±4.1
Constructional Apraxia - immediate copy	8	9.0±1.9
Words list - recall	4	5.5±2.2
Words list - recognition	8	9.0±1.7
Constructional Apraxia - recall	3	6.0±3.3
CFT	impaired	
Gesture Imitation	impaired	
Clock Completion Test	7 errors	0-3 errors
TMT-A	98 s	%<10
TMT-B	>302 s (NC)	%<10
TMT-B/TMT-A	>3	<3
CDR	1	0

ND: normative data; MMSE: Mini-Mental State Examination; CDR: Clinical Dementia Rating; CFT: Complex Figure Copying Test; TMT: Trail-Making Tests; CFT: Complex Figure Copying Test; NC: Task not completed; s: seconds; TMT-B/TMT-A ≥3 (impaired cognitive flexibility).

**Figure 1:**
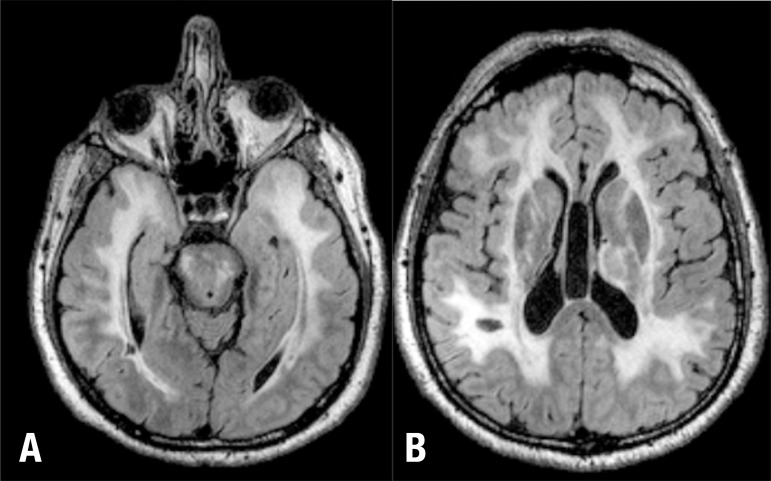
MRI FLAIR sequence in axial plan at temporal lobe [A] and basal ganglia [B] levels.

The patient was submitted to MRI which revealed, on FLAIR, extensive areas of hypersignal in subcortical white matter, predominantly frontal, temporal and parietal, in addition to compromised external and internal capsules, brain stem and presenting lacunar infarcts in the temporal and right parietal regions (Figure 1).

Morphometric analysis was also performed using segmentation by the signal intensity technique, evidencing the percentage of frontal lobe lesions (41.8%) (Table 2).

**Table 2:** Calculation of hyperintense brain lesion volume on FLAIR sequence.

Total Volume	% Total	FL	% Total	OL	% Total	PL	% Total	TL	% Total	Sum	% Dif.
287.0	100.0	119.9	41.8	3.8	1.3	26.7	9.3	57.4	20.0	207.8	27.6

Total volume: total volume of lesions in each lobe; %Total: percent of total volume of lesions; FL: frontal lobe; OL: occipital lobe; PL: parietal lobe; TL: temporal lobe; Sum: sum of lesions in each lobe; %Dif: percent of lesions out of the shown areas.

Genetic analysis was carried out (Laboratoire Génétique Moléculaire de l' Hôpital Lariboisière - Paris, Prof. Elisabeth Tournier-Lasserve) based on the direct DNA sequencing of exons 3 and 4 of the Notch 3 gene (chromosome 19), which revealed a nucleotide substitution of Arginine (CGC) to Cysteine (TGC) at position 153 in exon 4 (c.535 C >T: R153C), consistent with CADASIL diagnosis, confirming the etiology of the disease. Three of his siblings were later confirmed as carrying the same mutation. Recently, these individuals were included in a study assessing cognitive and neuroimaging profile.^15^

## DISCUSSION

Four large studies encompassing a total of 175 individuals have investigated the profile of cognitive decline in CADASIL.^16,17^ Of these studies, two focused on the relationship of the age effect and disease stage with cognitive profile.^17,18^

In the present case, changes were evident in global performance (MMSE) and in language, memory, apraxia and executive function domains.

In the language domain, both semantic verbal fluency (animals category) and naming ability were compromised. Deficit in verbal fluency is frequently observed in studies on CADASIL.^16,19^ In the study by Buffon et. al.,^18^ verbal fluency (semantic category) was found to be reduced.

Memory showed compromised register/learning yet better performance for recognition compared to spontaneous recall. Memory in patients with CADASIL tends to by relatively preserved, where patients may present compromise in immediate memory and free recall. On the other hand, both recall with cues and recognition are invariably preserved, suggesting that the encoding process is preserved.^16,19^

Results on the Complex Figure Copying and Gesture Imitation tests revealed the presence of constructional and ideomotor apraxia, respectively. This finding may be of particular importance given that it has been little discussed in the specialized literature. Some studies^20^ have reported ideomotor apraxia in 15% of individuals with lesions confined to the thalamic or lenticular region. Ragno et. al.,^21^ studied 12 individuals from two families and found that only one had deficit in ideomotor apraxia. Trojano et. al.,^22^ suggested that constructional and ideomotor apraxia can appear in some patients with cortical lesions. Peter et al.,^23^ in search of evidence, carried out a meta-analysis of reports published in the literature between 1994 and 1996, which included 82 patients and focused on apraxias associated with lesions in deep brain structures, such as the basal ganglia, thalamus and internal capsule. The study revealed that lesions to periventricular deep white matter play a crucial role in the development of apraxias, particularly ideomotor.

Executive function was also impaired, evidenced by reduced verbal fluency, planning difficulties and problems in space usage on the Clock Completion Test, slowness on the TMT-A (also reflecting attention deficit), incomplete TMT-B (also reflecting deficit in shifts in attention) and TMT-B/TMT-A >3, suggesting impaired cognitive flexibility. In line with findings of previous studies, executive dysfunction was clearly evident. Buffon et al.,^18^ in a study of 42 individuals with CADASIL, found executive dysfunction in almost 90% of individuals under 50 years of age, and suggested this finding may be explained by a decline in attention and memory performance consistent with some degree of frontal subcortical dysfunction.

Despite concerted research efforts, the mechanisms underlying cognitive dysfunction in CADASIL remain unclear. However, evidence suggests these mechanisms may be related to disruption of corticosubcortical/or corticocortical connections due to progressive damage to white matter^18^ and that cognitive decline in CADASIL is likely related to accumulated lacunar infarcts and augmented ventricular volume, but not to brain atrophy^24,25^.

**Conclusion.** The CADASIL case reported, in addition to exhibiting a characteristic neuroimaging pattern, was diagnostically confirmed by Notch-3 gene analysis. The neuropsychological findings were consistent with those reported in the literature, most notably the presence of apraxias, seldom mentioned in the specialized literature.

It is hoped that this individual and the other members of this and other families can benefit from the future development of protocols for pharmacological intervention and cognitive rehabilitation.

## References

[1] Joutel A, Corpechot C, Ducros A (1996). Notch3 mutations in CADASIL, a hereditary adult-onset condition
causing stroke and dementia. Nature.

[2] Chabriat H, Vahedi K, Iba-Zizen MT (1995). Clinical spectrum of CADASIL: a study of 7 families. Cerebral
autosomal dominant arteriopathy with subcortical infarcts and
leukoencephalopathy. Lancet.

[3] Tournier-Lasserve E, Joutel A, Melki J (1993). Cerebral autosomal dominant arteriopathy with subcortical
infarcts and leukoencephalopathy maps to chromosome 19q12. Nat Genet.

[4] Chabriat H, Levy C, Taillia H (1998). Patterns of MRI lesions in CADASIL. Neurology.

[5] Markus HS, Martin RJ, Simpson MA, Dong YB, Ali N, Crosby AH (2002). Diagnostic strategies in CADASIL. Neurology.

[6] Tomimoto H, Ohtani R, Wakita H, Lin JX, Miki Y, Mizuno T (2005). Distribution of ischemic leukoaraiosis in MRI: a difference from
white matter lesions in CADASIL. No To Shinkei.

[7] Schultz A, Santoianni R, Hewan-Lowe K (1999). Vasculopathic changes of CADASIL can be focal in skin
biopsies. Ultrastruct Pathol.

[8] Morris JC, Heyman A, Mohs RC (1989). The Consortium to Establish a Registry for Alzheimer's Disease
(CERAD). Part I. Clinical and neuropsychological assessment of Alzheimer's
disease. Neurology.

[9] Bertolucci PHF, Okamoto IH, Toniolo Neto J, Ramos LR, Brucki SMD (1998). Desempenho da população brasileira na bateria
neuropsicológica do Consortion to Establish a Registry for
Alzheimer´s Disease (CERAD). Rev Psiq Clin.

[10] Reitan RM, Wolfson D (1993). The Halstead-Reitan neuropsychological test battery: theory and clinical
interpretation.

[11] Watson YI, Arfken CL, Birge SJ (1993). Clock completion: an objective screening test for
dementia. J Am Geriatr Soc.

[12] Barbize J, Duizabo P (1985). Manual de Neuropsicologia.

[13] Morris JC (1993). The Clinical Dementia Rating (CDR): current version and scoring
rules. Neurology.

[14] Montano MB, Ramos LR (2005). Validity of the Portuguese version of Clinical Dementia
Rating. Rev Saude Publica.

[15] Silva JCV, Gasparetto Emerson L., André C (2011). Cognitive and neuroimaging profile of a Brazilian family with
CADASIL. Arq Neuropsiquiatr.

[16] Charlton RA, Morris RG, Nitkunan A, Markus HS (2006). The cognitive profiles of CADASIL and sporadic small vessel
disease. Neurology.

[17] Amberla K, Waljas M, Tuominen S, Almkvist O, Poyhonen M, Tuisku S, Kalimo H, Viitanen M (2004). Insidious cognitive decline in CADASIL. Stroke.

[18] Buffon F, Porcher R, Hernandez K, Kurtz A, Pointeau S, Vahedi K (2006). Cognitive profile in CADASIL. J Neurol Neurosurg Psychiatry.

[19] Peters N, Opherk C, Danek A, Ballard C, Herzog J, Dichgans M (2005). The pattern of cognitive performance in CADASIL - a monogenic
condition leading to subcortical ischemic vascular dementia. Am J Psych.

[20] Basso A, Faglioni P, Luzzatti C, Roy EA (1985). Methods in neuroanatomical research and experimental study of
limb apraxia. Neuropsychological studies of apraxia and related disorders.

[21] Ragno M, Fabrizi GM, Cacchiò G (2006). Two novel Italian CADASIL families from Central Italy with
mutation CGC-TGC at codon 1006 in the exon 19 Notch3 gene. Neurol Sci.

[22] Trojano L, Ragno M, Manca A, Caruso G (1998). A kindred affected by cerebral autosomal dominant arteriopathy
with subcortical infarcts and leukoencephalopathy (CADASIL). A 2-year
neuropsychological follow-up. J Neurol.

[23] Pramstaller Peter P., Marsden C. David (1996). The basal ganglia and apraxia. Brain.

[24] Yousry TA, Seelos K, Mayer M (1999). Characteristic MR lesion pattern and correlation of T1 and T2
lesion volume with neurologic and neuropsychological findings in cerebral
autosomal dominant arteriopathy with subcortical infarcts and
leukoencephalopathy (CADASIL). AJNR Am J Neuroradiol.

[25] Liem MK, Lesnik Oberstein SA, Haan J (2009). MRI correlates of cognitive decline in CADASIL: a 7-year
follow-up study. Neurology.

